# Modelling of n-Hexadecane bioremediation from soil by slurry bioreactors using artificial neural network method

**DOI:** 10.1038/s41598-022-21996-6

**Published:** 2022-11-16

**Authors:** Roya Morovati, Fariba Abbasi, Mohammad Reza Samaei, Hamid Mehrazmay, Ali Rasti Lari

**Affiliations:** 1 Department of Environmental Health Engineering, School of Health, Shiraz University of Medical Sciences, Shiraz, Iran; 2 Research Center for Health Sciences, Department of Environmental Health Engineering, School of Health, Shiraz University of Medical Sciences, Shiraz, Iran; 3 Bachelor of Engineering (Honours) in Environmental Engineering, School of Civil and Environmental Engineering, University of New South Wales, Sydney, NSW Australia; 4 Bachelor of Engineering (Honours) in Civil Engineering, School of Civil and Environmental Engineering, University of Technology Sydney, Sydney, NSW Australia

**Keywords:** Biological techniques, Microbiology, Environmental sciences, Solid Earth sciences

## Abstract

Diesel oil is known to be one of the major petroleum products that can pollute water and soil. Soil pollution caused by petroleum hydrocarbons has substantially impacted the environment, especially in the Middle East. In this study, modeling and optimization of hexadecane removal from soil was performed using two pure cultures of *Acinetobacter* and *Acromobacter* and consortium culture of both bacterial species using artificial neural network (ANN) method. Then the best ANN structure was proposed based on mean square error (MSE) as well as correlation coefficient (R) for pure cultures of *Acinetobacter* and *Acromobacter* as well as their consortium. The results showed that the correlations between the actual data and the data predicted by ANN (R2) in *Acromobacter*, *Acinetobacter* and consortium of both cultures were 0.50, 0.47 and 0.63, respectively. Despite the low correlation between the experimental data and the data predicted by the ANN, the correlation coefficient and the precision of ANN for the consortium was higher. As a result, ANN had desirable precision to predict hexadecan removal by the cobsertium culture of *Ochromobater* and *Acintobacter*.

## Introduction

Soil pollution caused by petroleum hydrocarbons is considered a major threat to the environment, especially in Middle Eastern countries. Failure in transmission lines, leakages from storage tanks and, oil tankers’ accidents are some of examples of soil pollution caused by petroleum hydrocarbons in Middle Eastern countries^1^. Diesel oil is an important crude oil product that has the tendency to pollute soil and water. It is produced during the oil refining process and consists of aromatic compounds, natural and, branched alkanes^2^. Among medium-chain alkanes, hexadecane (C_16_H_34_) has been studied as a model contaminant by many researchers^3–5^. Thus, treatment approaches to reduce the hazardous effects of hexadecane pollution are needed. Hexadecane is removed from soil and water by various methods, such as physical, thermal, chemical and, biological methods. Despite their low solubility in water, they are rapidly degraded by microorganisms^6^. Biological methods are one of the most common methods in decomposition and removal of these substances^7,8^. In addition to its simplicity, cost effectiveness and feasibility, it is also environmentally friendly recently, researchers have sought to optimize this process and utilize it ^3,9–11^. However, conventional physical–chemical treatments have high costs and can generate residues that are toxic to the biota^12^. Applying high efficiency and low cost bioremediation processes represent an extremely important way of recovering contaminated areas among several other clean up techniques. Treatment of soil in slurry bioreactors has gotten to be one of the leading alternatives for the bioremediation of soils contaminated by obstinate poisons beneath controlled natural conditions^13^. SBs are very often practical to determine the possibility and actual potential of a biological strategy in the final repair of a contaminated soil or site. In reality, beneath slurry conditions, the poison exhaustion rates depend primarily on the corruption action of the microorganisms accessible within the framework^14^ and the comes about gotten for the most part reflects the real natural depuration potential of the soil^13^. Modeling is an important tool for designing and operating a wastewater treatment process. To model wastewater treatment processes, various models such as Principal Component Analysis (PCA)^15,16^, Multiple Linear Regression (MLR)^17^, Random Forest (RF)^18,19^ and, Artificial Neural Networks (ANN)^20,21^ are implemented. Among these methods, ANN is a powerful method for modeling nonlinear systems particularly^22^.

Numerous researchers have examined the secondary effects of pollutants from simple assessable soil properties. Artificial neural network (ANN) models have been substituted in recent times for multiple linear regression (MLR) in developing prediction simulations for soil pollutants^23^. The major improvement of the ANN is that the models are trained to comprehend the non-linear and complex relationship between the input and output configurations and they do not put limitations on either the input or the output space^24^.

A major benefit of ANNs is their ability to detect trends in data that show significant unpredictable non-linearity. As a data-driven approach, The ANN may capture spatial features of the configuration at different scales describing linear and non-linear effects. Due to its simplicity in simulation, forecasting and, modeling, it is considered a promising tool^25^.

ANNs have been widely used in the last decade in forecast removing pollutants from the environment, thanks to their reliable, robust and salient characteristics of nonlinear relationships between input and output data. Nowadays, many researchers investigate these models and determine their ability to predict the bioremediation process, Bioremediation is a biological process that remediates the environment through processes alike adsorption, redox transformation, and precipitation reactions^26^. Due to the complex nature of the biodegradation process, it is difficult to modeled and simulated these processes by traditional mathematical models. Artificial neural networks (ANNs) are a promising and powerful modeling technique since the mathematical details of the phenomena related to the process are not required.

In this study 7 independent parameters (include the initial concentration of hexadecane, micronutrients concentration, C: N: P ratio, salt, seed, slurry and, exposure time of bacteria to the contaminant) were investigated on removal of hexadecane , and Since that the effects and relationship between parameters cannot be determined with linear relationships, In the present study, ANN models were developed to predict hexadecane removal efficiency from soil was performed using two pure cultures of *Acinetobacter* and *Acromobacter* and consortium culture of both bacterial species. , In the end, kinetic and linear equations have been used to check the factors on the removal efficiency.

## Materials and methods

### Laboratory activities

In this study, experimental data used for the removal of hexadecane from soil by slurry bioreactors using a combination of bioaugmentation and biostimulation. The experiment designed according to Taguchi’s method for optimizing the removal rate of hexadecane in the soil. The ratio depends on the quality characteristics of the product/process to be optimize. Taguchi's method uses the logarithmic functions to measure the signal-to-noise ratios (S/N). This technique is a powerful and high quality tool for the design of systems based on orthogonal array experiments that arrange the optimum setting of process control parameters. Moreover, it used as for the design of optimization step and output to serve as objective functions for optimization^27^. Besides, the designed processes and products by this method not influenced by external conditions^28^. Therefore, the noise decreased as the precision improved.

**Figure 1:**
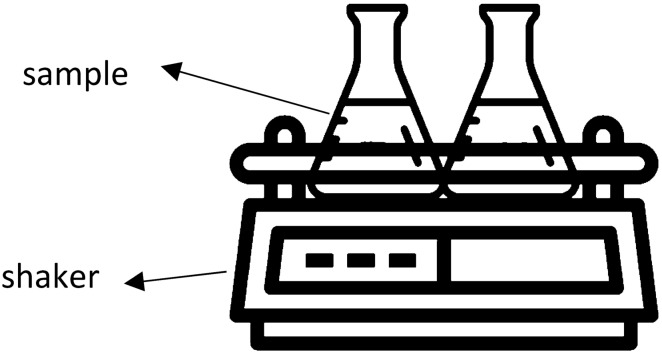
The schematic from this reactor.

First, the removal efficiency of biological method by in the slurry reactor carried out on 54 samples by Acinetobacter, Acromobacter and consortium mass. Then it compare to control samples that there is any bacteria in slurry. Moreover, the chemical properties of raw and effluent of reactor estimated that included pH, C:N:P, as well as the nutrients, salt and seed concentration. Reactor designed with dimension of 1 litter that clean soil was passed through a 2 mm sieve and washed with hexane, and dried. Then, it was spiked with hexadecane at a concentration of 3000 mg/kg dry soil. Then it well mixed completely and stored at 50 °C. After this, the soil seed by bacteria mass and finally it added to reactor for bioremediation^29^. The schematic from this reactor was shown in Fig. 1.

The pH of all bioreactors were adjusted between 7.4 and 7.8. It should be noted that all the experiments of this study were conducted in triplicate. The residual hexadecane was analyzed by GC-FID (Varian CP-3800, Palo Alto, CA, USA) after 20, 50 and 80 days.

### Design study

In this study, the rate of hexadecane removal from the soil investigated using two pure cultures of *Acinetobacter*, *Acromobacter*, and, a consortium culture of both bacterial species. All of bacteria mass cultured in Mineral salts medium (MSM) and trace elements included NH4Cl (4 g/L), KH2PO4 (2.5 g/L), NaCl (0.5 g/L), MgSO4 (0.3 g/L), FeCl3.6H2O (0.3 g/L), CaCl2 (0.01 g/L) and MnCl2.4H2O (0.01 g/L). MSM supported by micronutrients included MnCl2.4H2O (40 mg/L), MoO3 (80 mg/L), CuSO4 (6 mg/L), ZnSO4 (60 mg/L) and H3BO3 (0.03 mg/L). Furthermore, the effective variables of the experiment were also studied. These variables include the initial concentration of hexadecane, micronutrients concentration, C: N: P ratio, salt (NaCl = 0–1%), seed (active bacteria = 3–10% V nutrient broth with active bacteria to volume of bioreactor), slurry (soil/water ratio) and, exposure time of bacteria to the contaminant.

The total number of experiments for each culture was 30 runs. The range of each parameter is given in Table 1.

**Table 1:** The design study.

Variables	Level			Unite	
	1	2	3		
Initial hexadecane concentration	30	50	70	g / kg_soil_	
Nutrients ratio	0	2.50	5	% volume	
C:N:P ration	0.5:5:100	1:5:100	1:10:100	Percent	
Salt ratio	0	0.50	1	% volume	
Seed ratio	2.50	5	10	% volume	
Slurry ratio	5	10	20	g soil/L water	
Time exposure	10	50	80	Day	

### Analytical methods

#### ANN modelling

ANN is a powerful modeling method for determining the nonlinear relationship between variables^30^. This study was performed by feed-forward backpropagation and Levenberg–Marquardt using MATLAB 2018^31,32^. In the neural network, there are several inputs, hidden and output layers. The number of neurons in each hidden layer has very important in the response database. Hexadecane was predicted using two Multi-Layered Perceptrons (MLPs). Sigmoid and linear transfer functions were used for hidden and output layers, respectively. Model inputs included reaction time, initial hexadecane concentration, salt concentration, applied micronutrients concentration, reactor seeding rate, slurry percentage and, carbon to nitrogen to phosphorus ratio. In addition, the assigned weight determined the relationship between the layers.

Initially, the network was trained with laboratory data from cell viability, of which 70%, 15% and 15% of the data were used for training, validation and testing, respectively. Then, the weight and bias were determined based on network training to monitor the level of error validation. On the condition of an increase in validation error due to specific repetition, the network training was stopped to prevent overfitting. Therefore, it prevents from over fitting when the validation error increased with a specific epoch.

Due to the importance and accuracy of Mean Square Error (MSE) and correlation coefficient (R), the best ANN structure was selected based on MSE and R that were obtained by the number of neurons in the hidden layer. Therefore, the hexadecane removal percentage was predicted by pure cultures of both *Acinetobacter* and *Acromobacter* as well as their consortium.

The number of neurons in the hidden layer is determined based on the overlapping range of both Eqs. 1 and 2^33^.

1 $$\frac{2(i+o)}{3}<n<i\left(i+o\right)-1$$

2 $$0.5i - 2{\text{ < }}n{\text{ < }}2i + 2$$

i = number of inputs, o = the number of outputs and n = number of hidden layer neuron.

### Statistical analysis

According to this study, descriptive statistics such as mean, standard deviation, percentage and coefficient of variation have been used to indicate the concentration and hexadecane removal efficiency using the biostimulation method. Furthermore, analytical statistics, including t-test, was used to compare the hexadecane removal rate by different bacterial species under various conditions. The significance level was considered 0.05. Linear and nonlinear models, having different degrees and, weights were used to determine the effect of different variables on the hexadecane removal and the relationship between them. Finally, among the studied models included Polynominal, cubic interpolant, general model, Gussian, spline interpolant and quadratic regression, the best model was selected based on the correlation coefficient (R^2^) and the sum of squares errors (RMSE). In this study, MATLAB version 2018 software was used for modeling and Excel version 2013 software was used for graphs.

## Result and discussion

### Performance of slurry reactor in N-hexadecane removal using Acromobacter, Acinetobacter and their consortium bacteria

The hexadecane removal efficiency using slurry reactor by bacterial species including *Acromobacter*, *Acinetobacter* and, their consortium is shown in Table 4. The mean hexadecane removal by *Acinetobacter*, *Acromobacter* and, consortium of both species was 26.9 ± 9.9, 27.86 ± 11.98 and 27.94 ± 12.22 mg/L, respectively. According to Table 4, the maximum removal by *Acinetobacter* was 46.7% after 80 days of exposure. In these conditions, the concentration of nutrients, salt, inoculation, C: N: P ratio and slurry percentage to 2.5, 0.5, 10, 4.17 and 5%, respectively. Also, the maximum in vitro *Acromobacter* removal was 59.2%, which at the initial concentration of 30 mg / L, micronutrient equal to 2.5%, slurry and seed content equal to 20 and 2.5% respectively, and carbon to nitrogen ratio 4.17 without salt is obtained. The maximum removal percentage by bacterial consortium, having a concentration of 50 mg / l, resulted in the following percentages: 2.5% of micronutrients, 1% of salt, 2.5% of seed and 5% of slurry. Additionally, carbon to nitrogen ratio of 4.17 was obtained in a period of 50 days. Because the mineralization of the petroleum compounds was performed by consortium cultures at higher concentrations. So that metabolites of one species are used as substrate for other species^34^. In this condition, the inhibitory effect of metabolites and high concentrations of initial substrate was reduced.

### Prediction of hexadecane removal efficiency using artificial neural network

Another concept studied in this research paper was the ability of the neural networks to predict hexadecane removal using *Acinetobacter*, *Acromobacter* and, consortium bacteria of both bacterial species. They were investigated separately using the several algorithms that the best results was associated to levenberg-marque (Table 2).

**Table 2:** The results of other algorithms.

Training algorithm	Rall			Rtest			MSE			MAE		
	O	A	C	O	A	C	O	A	C	O	A	C
Conjugate gradient with powell-beal rastarts	0.79	0.64	0.68	0.89	0.92	0.83	38	32	10	45	36	11.5
Gradient Descent	0.81	0.68	0.7	0.91	0.91	0.82	41	31	11.2	41	37	12
Levenberg–Marquardt	0.85	0.7	0.72	0.94	0.95	0.84	38	31	9.5	41	35	11.4
Scaled Conjugate gradient	0.83	0.63	0.69	0.87	0.89	0.81	38	34	10.5	43	37	11.7
Backpropagation	0.74	0.66	0.71	0.9	0.91	0.79	39	33	11	42	36	11.9

Based on Table 2, the best results associated to Levenberg–Marquardt that expressed in article.

**Figure 2:**
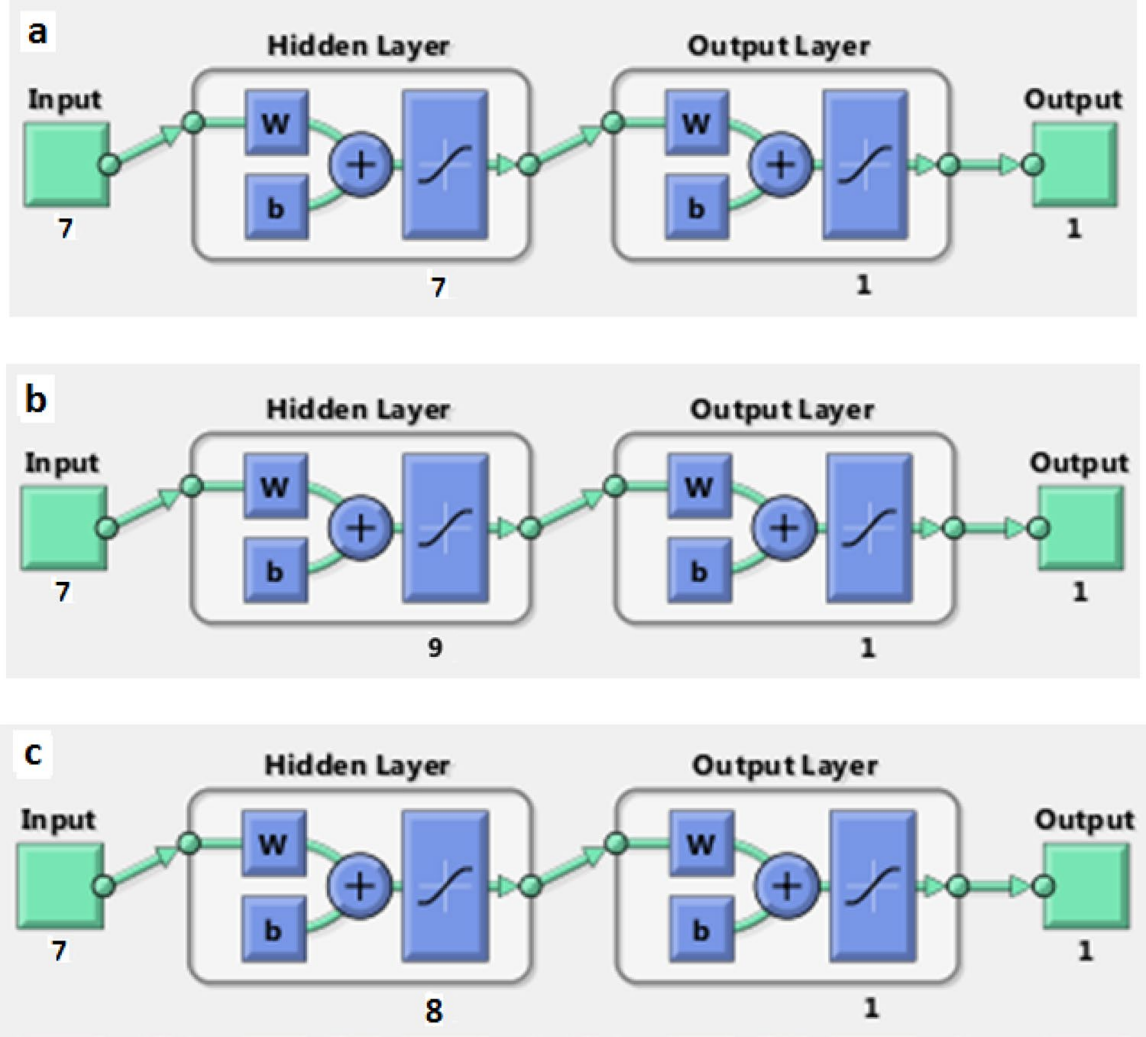
The plot of optimum structure of ANN. (**a**) *Acintobacter*, (**b**) *Acromobacter* and (**c**) consortium.

Then the results from this algorithm with a number of different neurons in the hidden layer were estimated. Based on the overlapping range of Eqs. (1) and (2) and according to the number of input and output variables, 6–16 neurons were placed in the hidden layer. Based on the MSE rate and correlation coefficient, the best model was selected. The plot of the best model for all bacteria culture and its results are shown in Fig. 2 and Table 3.

**Table 3:** Performance of the artificial neural network during changes in the number of neurons in the lattice.

Structure	*Acintobacter*					*Acromobacter*					*Consortium*				
	R all	R test	R valid	MSE	MAOP	R all	R test	R valid	MSE	MAOP	R all	R test	R valid	MSE	MAOP
7:6:1	0.67	0.94	0.76	39.59	45	0.69	0.81	0.91	40.58	44.8	0.67	0.82	0.82	16.90	10.9
7:7:1	**0.75**	**0.96**	**0.81**	**32.90**	**40.5**	0.68	0.85	0.86	53.60	45.6	0.73	0.84	0.88	18.60	11.3
7:8:1	0.64	0.93	0.78	51.9	42.4	0.73	0.89	0.9	57.90	46	**0.89**	**0.85**	**0.89**	**9.44**	**10.4**
7:9:1	0.66	0.95	079	78	44	**0.89**	**0.98**	**0.94**	**40.50**	**42**	0.75	0.81	0.87	30.40	11.4
7:10:1	0.68	0.87	0.73	20.99	46	0.76	0.83	0.92	43.50	43.5	0.78	0.83	0.85	12.70	12.7
7:11:1	0.60	0.78	0.68	47.7	45.3	0.74	0.87	0.87	58.70	44.6	0.64	0.79	0.86	18.40	11.8
7:12:1	0.67	0.81	0.74	0.14	46.2	0.69	0.88	0.89	63.60	43.9	0.68	0.78	0.88	13.50	10.8
7:13:1	0.63	0.74	0.78	48.20	45.8	0.74	0.85	0.94	78.40	45.2	0.71	0.8	0.84	4.30	11.4
7:14:1	0.52	0.62	0.73	77.13	44.9	0.71	0.86	0.83	96.94	44.7	0.68	0.76	0.83	46.70	10.75
7:15:1	0.63	0.72	0.8	68.80	43.8	0.64	0.89	0.89	85.30	43.8	0.76	0.78	0.84	53.50	11.2
7:16:1	0.49	0.75	0.73	18.70	44.2	0.62	0.95	0.88	91.70	44.1	0.65	0.73	0.78	51.60	12.8

Significant values are in bold. Values are in bold (maximum and minimum response levels).

According to Table 3, the best ANN topology for predicting the removal of hexadecanes for *Acromobacter*, *Acinetobacter* and, their consortium was 7: 9: 1, 7: 7: 1 and 7: 8: 1, respectively. In addition, according to Fig. 1, the minimum ANN error for predicting the removal percentage for all three cultivars: *Acinetobacter*, *Acromobacter* and consortium was 40.5, 32.9 and 9.4, at epochs of 1, 5 and 4 respectively. Despite that, the increase rate of validation error was such that for all three cultures, the algorithm was stopped in iterations 7, 11 and 10, respectively. Besides, R_all_ (the total regression coefficient of ANN structure) for the hexadecane removal by *Acromobacter*, *Acinetobacter* and, their consortium was 0.75, 0.89 and 0.89, respectively. As the R for all of them were higher than 0.75, the prediction by using this model was good^35^. In other studies, R_all_ for PAH removal was higher than 0.9 using ANN^24,36^. According to these results, the ANN has a high tendency to predict hexadecane removal by using *Acromobacter* species. Table 3 shows the mean N-hexadecane removal by all three cultures in the actual data and the values predicted by ANN.

According to Table 4, the average hexadecane removal by consortium, *Acinetobacter* and, *Acromobacter* was 26.9%, 27.86% and 27.92%, respectively. Moreover, the best removal efficiency for any culture in real data was expressed in Table 5. Because it is expected that in consortium cultures with the production of more surfactants, more ester linkage is formed between hexadecane and biosurfactants, which is effective in reducing toxicity and increasing bioremediation^32,37^. The average elimination predicted in ANN by the consortium, *Acinetobacter* and *Acromobacter* was 26.94, 28.9 and 27.66%, respectively. The standard deviation and coefficient of variation predicted in ANN was lower than the actual data.

**Table 4:** Average removal of N-hexadecane by all three cultures in actual data and values predicted by ANN.

Removal of N-hexadecane	Actual data			ANN predicted		
	*Acintobacter*	*Acromobacter*	Consortium	*Acintobacter*	*Acromobacter*	Consortium
Average	26.90	27.86	27.92	26.94	28.90	27.66
SD	9.90	11.98	12.22	8.02	4.60	9.69
CV	1.50	1.80	1.47	1.13	1.05	1.42

**Table 5:** The design matrix of independent and dependent variables. a) *Acintobacter*, b) *Acromobacter* and c) consortium of *Acintobacter* and *Acromobacter*.

Runs	Hexan conc (gr/kg soil)	Nutrients	Salt (volume percent)	Seed percent	C:N:P	Slurry percent	Time (day)	Actual % removal	ANN predicted % removal
*a*									
1	30	0	0	2.5	5	5	10	29.42	30.16
2	50	2.5	0.5	10	4.17	5	10	26.93	24.63
3	70	2.5	1	5	5	20	10	14.78	31.98
4	30	0	1	10	4.17	10	10	15.31	**9.31**
5	50	5	0	5	4.17	10	10	**6.24**	27.88
6	70	5	0.5	2.5	4.17	20	10	17.83	18.15
7	30	0	0	2.5	5	5	20	32.57	33.79
8	50	2.5	0.5	10	4.17	5	20	28.61	29.80
9	70	2.5	1	5	5	20	20	38.82	38.34
10	30	0	1	10	4.17	10	20	10.63	12.59
11	50	5	0	5	4.17	10	20	29.02	28.98
12	70	5	0.5	2.5	4.17	20	20	25.59	19.94
13	30	0	0	2.5	5	5	50	40.90	36.81
14	50	2.5	0.5	10	4.17	5	50	32.48	36.1
15	70	2.5	1	5	5	20	50	25.41	21.77
16	30	0	1	10	4.17	10	50	32.31	32.22
17	50	5	0	5	4.17	10	50	21.71	27.81
18	70	5	0.5	2.5	4.17	20	50	12.26	20.56
19	30	0	0	2.5	5	5	50	32.68	36.81
20	50	2.5	0.5	10	4.17	5	50	44.12	36.12
21	70	2.5	1	5	5	20	50	17.32	21.77
22	30	0	1	10	4.17	10	50	33.09	32.22
23	50	5	0	5	4.17	10	50	33.11	27.81
24	70	5	0.5	2.5	4.17	20	50	36.24	20.56
25	30	0	0	2.5	5	5	80	31.17	30.83
26	50	2.5	0.5	10	4.17	5	80	**46.75**	**39.78**
27	70	2.5	1	5	5	20	80	17.89	17.63
28	30	0	1	10	4.17	10	80	19.32	22.95
29	50	5	0	5	4.17	10	80	29.50	26.43
30	70	5	0.5	2.5	4.17	20	80	24.98	14.50
*b*									
1	30	2.5	0.5	5	4.17	10	10	25.88	27.64
2	50	5	1	2.5	5	10	10	**8.88**	16.32
3	70	5	0	10	4.17	5	10	39.07	35.11
4	30	2.5	0	2.5	4.17	20	10	41.67	53.37
5	50	0	0.5	10	5	20	10	31.15	31.07
6	70	0	1	5	4.17	5	10	6.54	8.61
7	30	2.5	0.5	5	4.17	10	20	27.65	25.51
8	50	5	1	2.5	5	10	20	31.59	21.76
9	70	5	0	10	4.17	5	20	19.62	26.15
10	30	2.5	0	2.5	4.17	20	20	**59.22**	49.67
11	50	0	0.5	10	5	20	20	19.79	28.37
12	70	0	1	5	4.17	5	20	20.43	7.83
13	30	2.5	0.5	5	4.17	10	50	21.50	22.36
14	50	5	1	2.5	5	10	50	35.03	32.02
15	70	5	0	10	4.17	5	50	18.33	17.14
16	30	2.5	0	2.5	4.17	20	50	18.24	36.75
17	50	0	0.5	10	5	20	50	9.96	21.69
18	70	0	1	5	4.17	5	50	18.78	7.64
19	30	2.5	0.5	5	4.17	10	50	35.62	22.36
20	50	5	1	2.5	5	10	50	29.17	32.02
21	70	5	0	10	4.17	5	50	27.81	17.14
22	30	2.5	0	2.5	4.17	20	50	49.26	36.75
23	50	0	0.5	10	5	20	50	33.53	21.69
24	70	0	1	5	4.17	5	50	26.78	7.65
25	30	2.5	0.5	5	4.17	10	80	37.50	24.03
26	50	5	1	2.5	5	10	80	38.98	38.34
27	70	5	0	10	4.17	5	80	18.61	19.82
28	30	2.5	0	2.5	4.17	20	80	40.38	48.86
29	50	0	0.5	10	5	20	80	28.69	28.99
30	70	0	1	5	4.17	5	80	16.05	15.93
*c*									
1	30	5	1	10	4.17	20	10	24.33	21.31
2	50	0	0	5	4.17	20	10	34.06	27.22
3	70	0	0.5	2.5	4.17	10	10	**8.18**	17.39
4	30	5	0.5	5	5	5	10	17.79	21.93
5	50	2.5	1	2.5	4.17	5	10	35.76	28.34
6	70	2.5	0	10	5	10	10	3.86	**7.25**
7	30	5	1	10	4.17	20	20	27.85	33.70
8	50	0	0	5	4.17	20	20	20.59	26.88
9	70	0	0.5	2.5	4.17	10	20	30.61	19.60
10	30	5	0.5	5	5	5	20	33.18	24.23
11	50	2.5	1	2.5	4.17	5	20	34.99	34.45
12	70	2.5	0	10	5	10	20	11.85	11.76
13	30	5	1	10	4.17	20	50	46.98	45.67
14	50	0	0	5	4.17	20	50	23.11	24.65
15	70	0	0.5	2.5	4.17	10	50	22.38	20.79
16	30	5	0.5	5	5	5	50	42.94	33.31
17	50	2.5	1	2.5	4.17	5	50	22.47	34.04
18	70	2.5	0	10	5	10	50	9.79	18.69
19	30	5	1	10	4.17	20	50	48.60	45.67
20	50	0	0	5	4.17	20	50	30.44	24.65
21	70	0	0.5	2.5	4.17	10	50	27.86	20.79
22	30	5	0.5	5	5	5	50	32.84	33.31
23	50	2.5	1	2.5	4.17	5	50	**49.21**	34.04
24	70	2.5	0	10	5	10	50	26.25	18.69
25	30	5	1	10	4.17	20	80	47.81	**46.54**
26	50	0	0	5	4.17	20	80	41.57	41.41
27	70	0	0.5	2.5	4.17	10	80	15.81	33.88
28	30	5	0.5	5	5	5	80	21.31	23.51
29	50	2.5	1	2.5	4.17	5	80	24.90	32.96
30	70	2.5	0	10	5	10	80	20.14	23.20

Significant values are in [bold]. Values are in bold (maximum and minimum response levels).

According to Table 5, the percentage of hexadecane removal by *Acinetobacter* ranged between 6% up to 46%, while ANN predicted a removal range of 9–39%. Moreover, the lowest and highest percentages of hexadecane removal by *Acinetobacter* occurred in the 5th and 26th runs, respectively, whereas they related to runs 4 and 39 by ANN. Although during the actual conditions, the maximum *Acinetobacter* removal rate was in accordance with the ANN, the range of ANN changes in the consortium was more in line with the actual conditions. In other studies, it has been predicted that microbial populations with 4-9-1 and 3-25-1 structures were used for the removal of toluene and trichloroethylene. Therefore, the results of this study were confirmed by previous studies^30,31^.

**Figure 3:**
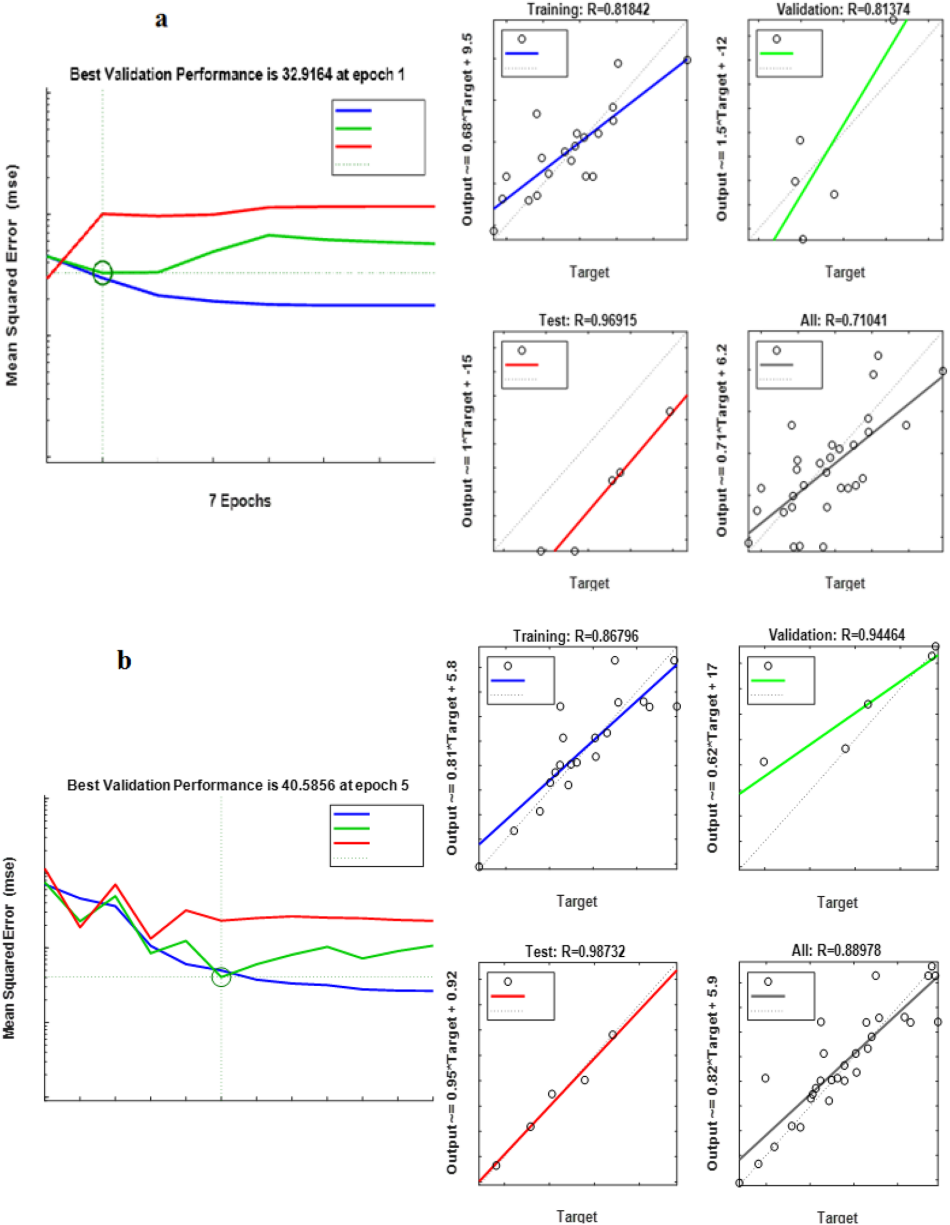

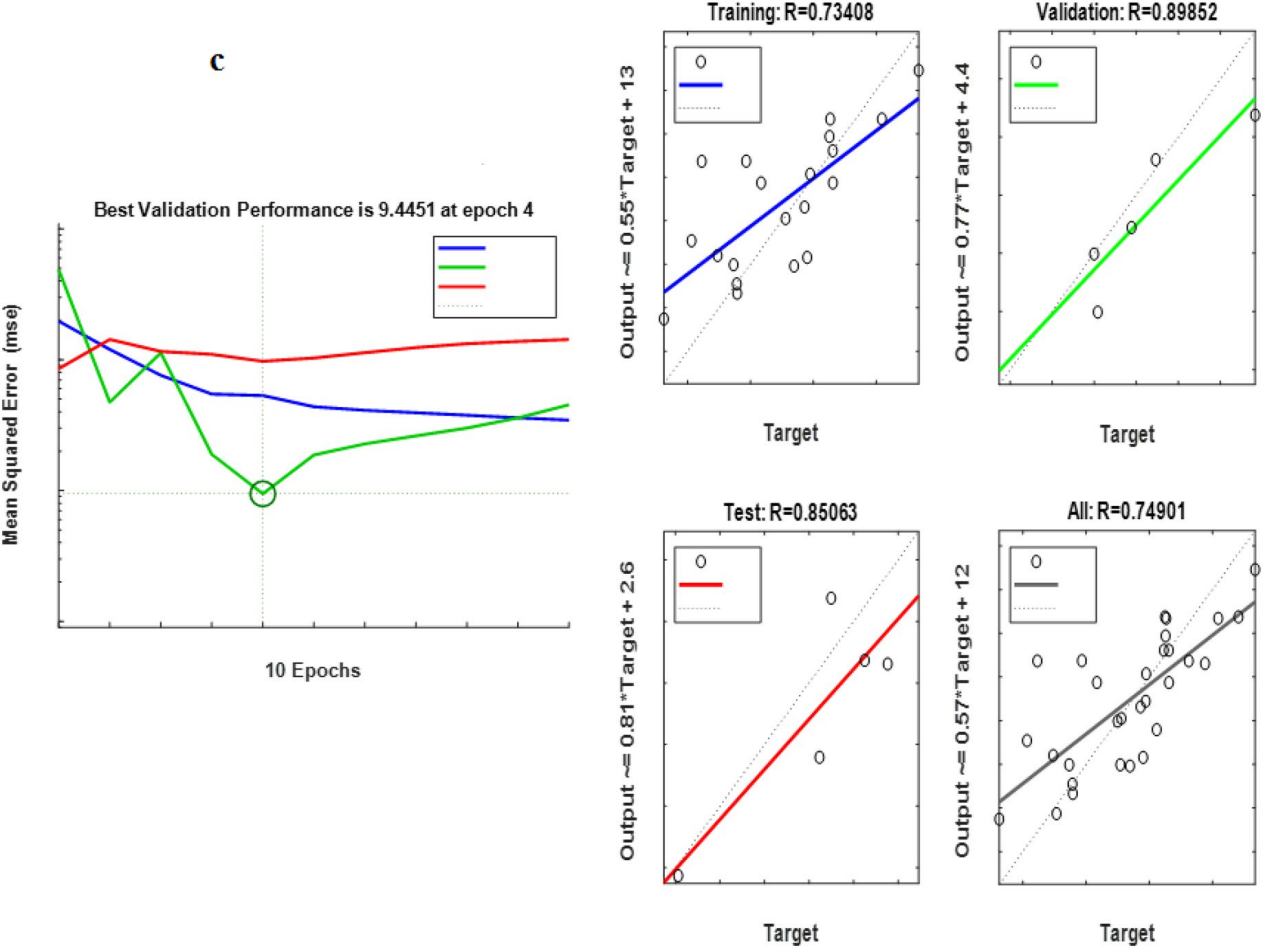
The performance of ANN. (**a**) *Acintobacter*, (**b**) *Acromobacter* and (**c**) consortium of *Acintobacter* and *Acromobacter*.

The correlation values predicted by ANN and actual data for all three culture are shown in Fig. 3.

**Figure 4:**
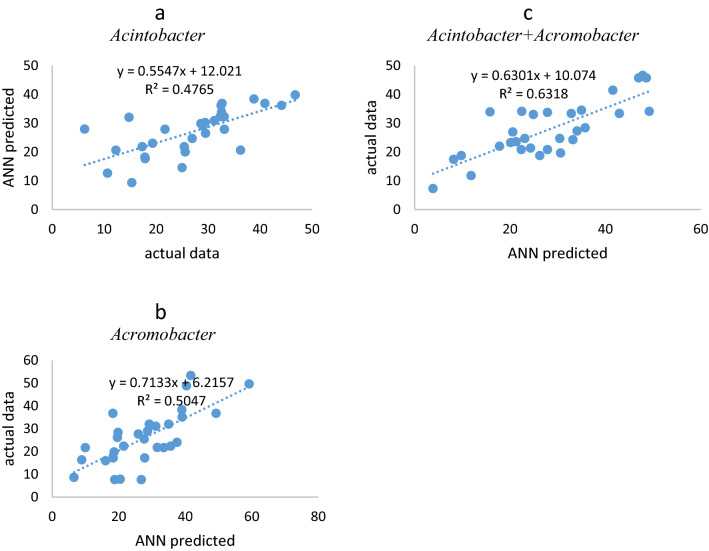
The correlation coefficient of ANN predicted vs actual data. (**a**) *Acintobacter*, (**b**) *Acromobacter* and (**c**) consortium of *Acintobacter* and *Acromobacter*.

According to Fig. 4, the correlation between the actual data and the data predicted by ANN (R^2^) in *Acromobacter*, *Acintobacter*, and Consortium of both crops was 0.50, 0.47 and 0.63, respectively. Although there was no high correlation between the actual data and the ANN forecast, the forecast was higher for the consortium. According to these results, ANN had a higher ability to predict hexadecane removal using *Acromobacter* and *Acinetobacter* consortium. Similar results were also indicated by previous studies^38,39^.

### The effect of independent variables on n-hexane removal during the exposure time and initial hexadecane concentration

**Figure 5:**
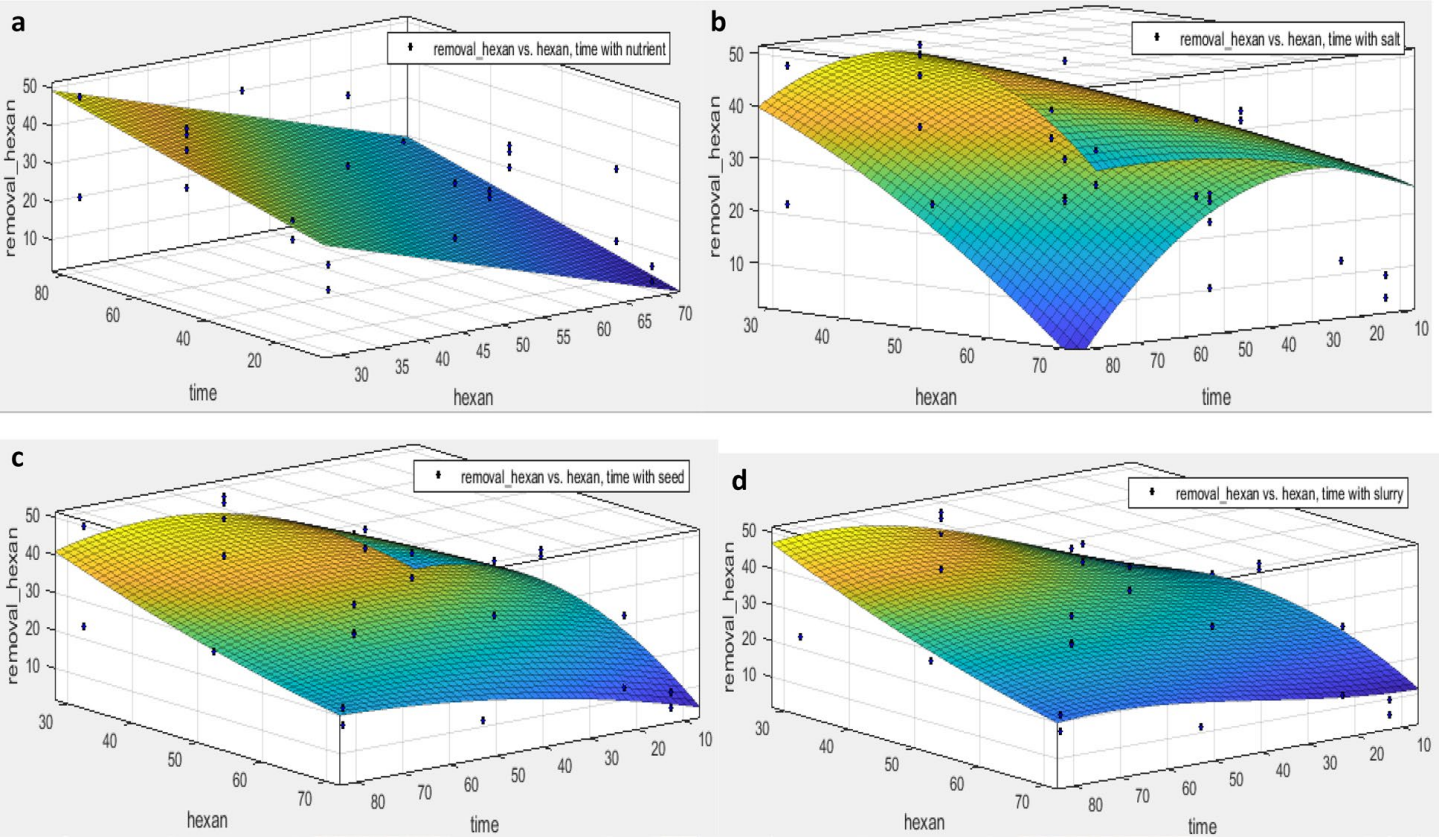
The effect of exposure time and initial hexane concentration on hexane removal perxent. (**a**) the presence of nutrient, (**b**) the presence of microbial salt, (**c**) the presence of seed and d) the presence of slurry, by excel version 2013 software.

As the concentration of pollutants continuously changes in the ambient environment, the synergistic effect both exposure time and initial concentration of hexadecane on the removal efficiency (actual data) predicted by the regression model (Fig. 5). Also, to compare the conditions predicted by ANN, the concentrations obtained from ANN are shown in Fig. 4.

**Figure 6:**
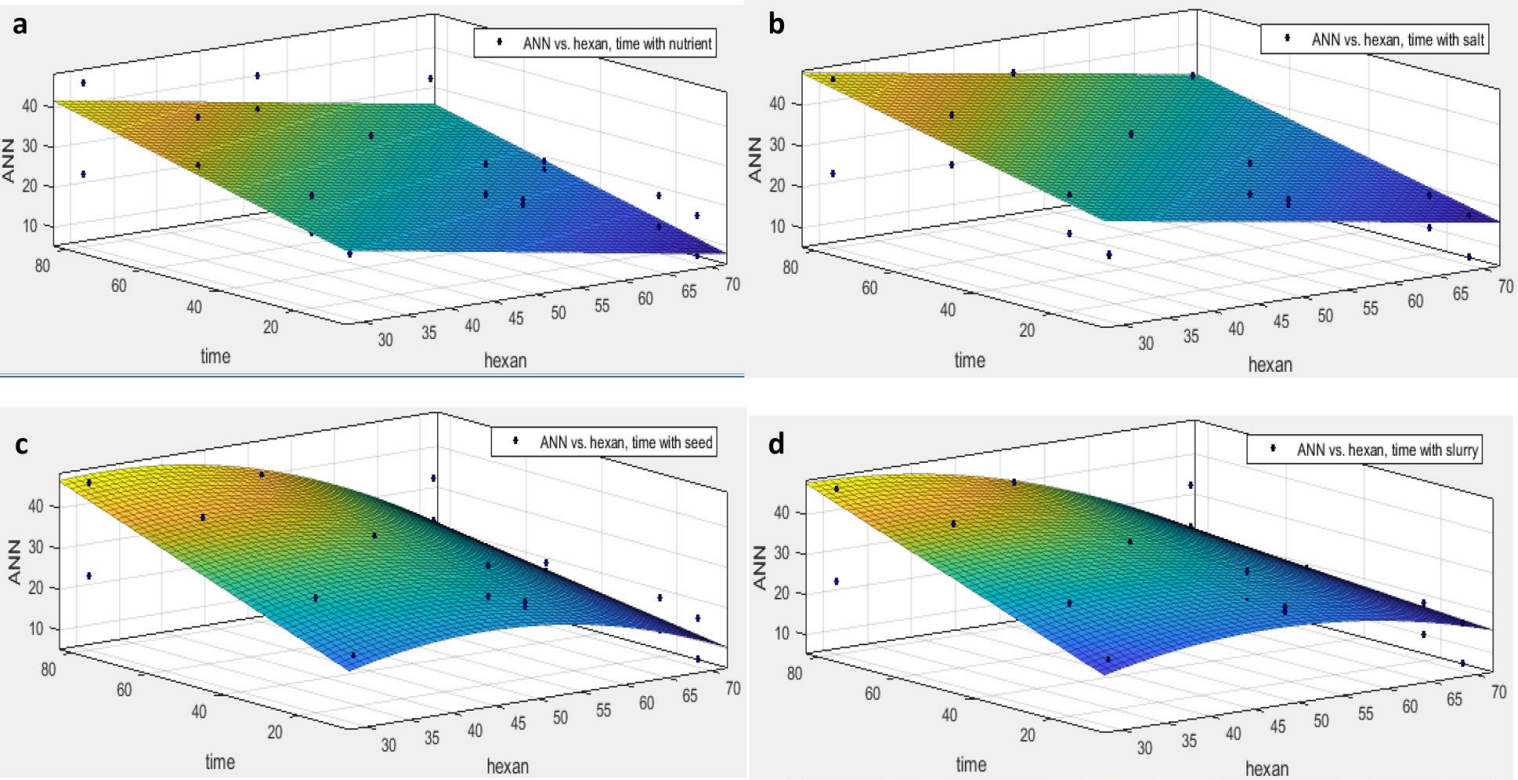
The effect of exposure time and initial hexane concentration on hexane removal perxent by ANN modeling. (**a**) the presence of nutrient, (**b**) the presence of microbial salt, (**c**) the presence of seed and d) the presence of slurry, by excel version 2013 software.

As shown in Fig. 6, the maximum removal rate of N-hexadecane occurred at a low concentration (30 g / kg) with an exposure time of 80 days. According to Table 6, considering the factors affecting the growth of consortium bacteria, the percentage of hexadecane removal in the presence of different concentrations of nutrients has followed the first-order equation. This is because the compound has a linear structure, and its mineralization rate depends on bioavailability^29^.

**Table 6:** The fitting of effective variables on removal of hexane during the variation of initial hexane concentration and time exposure of consortium culture.

	Effective variable	Regression equation	SSE	R2	Adjusted-R2	RMSE	Robust
Actual data	Nutrient	f(x,y) = 48.78 – 0.67 x + 0.23 y	1754	0.85	0.84	8.06	LAR
	Salt	f(x,y) = − 9.149 + 0.98 x + 1.62 y – 0.008 x^2^ – 0.013 × y – 0.01 y^2^	523.60	0.73	0.67	4.67	LAR
	Seed	f(x,y) =—43.78 + 2.84 x + 2.3 y – 0.03 x^2^ – 0.056 × y – 0.01 y^2^ + 0.0004 x^2^ y + 0.00014 × y^2^ – 0.000001 y^3^	9867	0.68	0.55	21.68	off
	Slurry	f(x,y) = − 18.3 + 1.9 x + 1.43 y – 0.021 x^2^ – 0.043 × y + 0.0008 y^2^ + 0.0003 x^2^ y + 0.00008 × y^2^ – 0.00006 y^3^	20,570	0.61	0.46	31.29	LAR
ANN predicted	Nutrient	f(x,y) = 32.68 – 0.36 x + 0.23 y	983	0.88	0.87	6.03	Off
	Salt	f(x,y) = 39.98 – 0.35 x + 0.2 y	147.60	0.87	0.86	2.34	Off
	Seed	f(x,y) = − 12.27 + 1.54 x + 0.48 y – 0.02 x^2^ – 0.005 × y	2867	0.87	0.85	10.71	Off
	Slurry	f(x,y) = − 10.06 + 1.376 x + 0.55 y – 0.014 x^2^ – 0.0067 × y	5196	0.86	0.84	14.42	Off

x = hexan, y = time.

While in the presence of different concentrations of salt, seed, and slurry; it fitted with the square of hexadecane concentration and the cube of exposure time. While the values predicted by ANN show that the removal percentage in the presence of different concentrations of nutrients and salt, it is better predicted by the first-order polynomial regression. However, in the presence of different concentrations of seed and slurry, the best fitting was provided with quadratic regression, as seen in Table 6.

In actual data, changes in micronutrient concentrations have been linearly associated with decreasing hexadecane concentrations and increasing exposure time. While for salt, seed and slurry only the removal percentage is optimized during a long reaction time and extremely low concentrations. In terms of cost–benefit assessments, these conditions are not appropriate.

Thus, it is better to achieve the desired conditions in studies or microbial decontamination by changing the concentration of micronutrients at lower costs. Meanwhile, the artificial neural network predicts similar nutrient conditions for salt. It seems that further studies on the salt parameter are necessary to determine the validity of the neural network.

In the modeling conducted by ANN, the best model was obtained by the simultaneous effect of slurry and salt with the polynomial regression model (SSE: 338.7, R-square: 0.8757, Adjusted R-square: 0.8665, RMSE: 3.542). This was due to the fact that both parameters in the actual deletion of hexadecane had less correlation with the polynomial regression model (SSE: 2363, R-square: 0.4551, Adjusted R-square: 0.3415, RMSE: 9.923) Figure 4.

On the other hand, the best suited model in terms of actual removal was related to the simultaneous effect of slurry and time by using the response surface (SSE: 1915, R-square: 0.5584, Adjusted R-square: 0.4664, RMSE: 8.933) (Table 6). However, according to Gosai et al. the neural network performed better than the linear model in predicting PAH removal^40^. This was due to hexadecane having a linear structure, thereby they are better predicted by linear models.

## Conclusions

In this study, the removal of N-hexadecane from soil was modeled by *Acromobacter*, *Acinetobacter* and their consortium in the slurry reactor by the neural network. Subsequently, the effect of functional parameters on biological removal was investigated using linear regression models. The modeling results showed that the artificial neural network had a higher ability in predicting the biological removal of N-hexadecane from the soil using the microbial population of *Acromobacter*. So that a higher correlation coefficient was obtained that there was no overfitting until the fifth epoch. In addition, the greater number of neurons in the lattice layer indicated that there was more complex non-linear relationships for prediction of hexadecane biodegradation using *Acromobater* than *Acintobacter*. On the other hand, in both, real data and neural network prediction, N-hexadecane removal was associated with first-degree nutritional concentration while it was associated with second-degree seed and slurry regression. However, different results have been obtained regarding time and salt concentration, suggesting that more studies should be done in this field. Furthermore, only the removal percentage is optimised during prolonged shelf life and low concentrations for salt, seed, and slurry, which is not suitable for cost–benefit assessments.

## Supplementary Information

Supplementary Information.

## Data Availability

All data generated or analysed during this study are included in this published article [and its supplementary information files].
