# Supplementary material for: Modelling of n-Hexadecane bioremediation from soil by slurry bioreactors using artificial neural network method
Source: Sci Rep. 2022 Nov 16;12:19662. doi: 10.1038/s41598-022-21996-6 (PMC9669037; doi:10.1038/s41598-022-21996-6)
Supplement: Supplementary file 1 — Supplementary Information. [file 41598_2022_21996_MOESM1_ESM.docx]

| Non Biologically  Hexadecane concentration(mg/l) | | | | |
| --- | --- | --- | --- | --- |
| Day 0 | 10 | 20 | 50 | 80 |
| 30 | 23.2155 | 18.20464 | 16.4035 | 15.77134 |
| 30 | 23.2155 | 18.20464 | 16.4035 | 15.77134 |
| 30 | 23.2155 | 18.20464 | 16.4035 | 15.77134 |
| 50 | 27.50122 | 23.87412 | 22.28731 | 24.72818 |
| 50 | 27.50122 | 23.87412 | 22.28731 | 24.72818 |
| 50 | 27.50122 | 23.87412 | 22.28731 | 24.72818 |
| 70 | 35.39647 | 35.15033 | 23.29066 | 18.68335 |
| 70 | 35.39647 | 35.15033 | 23.29066 | 18.68335 |
| 70 | 35.39647 | 35.15033 | 23.29066 | 18.68335 |
| 30 | 23.2155 | 18.20464 | 16.4035 | 15.77134 |
| 30 | 23.2155 | 18.20464 | 16.4035 | 15.77134 |
| 30 | 23.2155 | 18.20464 | 16.4035 | 15.77134 |
| 50 | 27.50122 | 23.87412 | 22.28731 | 24.72818 |
| 50 | 27.50122 | 23.87412 | 22.28731 | 24.72818 |
| 50 | 27.50122 | 23.87412 | 22.28731 | 24.72818 |
| 70 | 35.39647 | 35.15033 | 23.29066 | 18.68335 |
| 70 | 35.39647 | 35.15033 | 23.29066 | 18.68335 |
| 70 | 35.39647 | 35.15033 | 23.29066 | 18.68335 |
| Non Biologically Removal | | | | |
| Day 0 | 10 | 20 | 50 | 80 |
| 30 | 6.784496 | 11.79536 | 13.5965 | 14.22866 |
| 30 | 6.784496 | 11.79536 | 13.5965 | 14.22866 |
| 30 | 6.784496 | 11.79536 | 13.5965 | 14.22866 |
| 50 | 22.49878 | 26.12588 | 27.71269 | 25.27182 |
| 50 | 22.49878 | 26.12588 | 27.71269 | 25.27182 |
| 50 | 22.49878 | 26.12588 | 27.71269 | 25.27182 |
| 70 | 34.60353 | 34.84967 | 46.70934 | 51.31665 |
| 70 | 34.60353 | 34.84967 | 46.70934 | 51.31665 |
| 70 | 34.60353 | 34.84967 | 46.70934 | 51.31665 |
| 30 | 6.784496 | 11.79536 | 13.5965 | 14.22866 |
| 30 | 6.784496 | 11.79536 | 13.5965 | 14.22866 |
| 30 | 6.784496 | 11.79536 | 13.5965 | 14.22866 |
| 50 | 22.49878 | 26.12588 | 27.71269 | 25.27182 |
| 50 | 22.49878 | 26.12588 | 27.71269 | 25.27182 |
| 50 | 22.49878 | 26.12588 | 27.71269 | 25.27182 |
| 70 | 34.60353 | 34.84967 | 46.70934 | 51.31665 |
| 70 | 34.60353 | 34.84967 | 46.70934 | 51.31665 |
| 70 | 34.60353 | 34.84967 | 46.70934 | 51.31665 |
| Biologically Removal | | | | |
|  | 10 | 20 | 50 | 80 |
|  | 14.83715 | 11.47122 | 11.79238 | 8.809126 |
|  | 7.289086 | 9.410195 | 11.24551 | 11.41139 |
|  | 5.275958 | 4.643501 | 10.24991 | 7.457197 |
|  | 9.06459 | 11.50771 | 12.94668 | 17.11034 |
|  | 18.78084 | 16.09721 | 16.45301 | 17.81617 |
|  | 10.0508 | 15.3614 | 16.22731 | 19.17846 |
|  | 10.3112 | 21.11852 | 16.33093 | 15.30549 |
|  | 8.774507 | 4.497935 | 8.219369 | 9.377524 |
| All Removal% | | | | |
|  | 10 | 20 | 50 | 80 |
|  | 21.62165 | 23.26658 | 25.38888 | 23.03778 |
|  | 14.07358 | 21.20555 | 24.84201 | 25.64005 |
|  | 12.06045 | 16.43886 | 23.84641 | 21.68585 |
|  | 31.56337 | 37.63359 | 40.65937 | 42.38216 |
|  | 41.27962 | 42.2231 | 44.16571 | 43.08799 |
|  | 32.54957 | 41.48728 | 43.94 | 44.45029 |
|  | 44.91473 | 55.96818 | 63.04027 | 66.62215 |
|  | 43.37804 | 39.3476 | 54.9287 | 60.69418 |
